# Sleepwalking, sleep-related eating disorder and sleep-related smoking successfully treated with topiramate: a case report

**DOI:** 10.5935/1984-0063.20220065

**Published:** 2022

**Authors:** Sana Elham Kazi, Jamal Mujaddid M Mohammed, Carlos H Schenck

**Affiliations:** 1 Interfaith Medical Center, Department of Psychiatry - Brooklyn - New York - United States; 2 University of California Davis, Department of Internal Medicine - Sacramento - California - United States; 3 Northern California Veterans Affairs Health Care System, Sleep Medicine - Sacramento - California - United States; 4 Minnesota Regional Sleep Disorders Center, Sleep Medicine - Minneapolis - Minnesota - United States; 5 University of Minnesota Medical School, MD - Minneapolis - Minnesota - United States; 6 Hennepin County Medical Center, Department of Psychiatry - Minneapolis - Minnesota - United States

**Keywords:** Sleep-Related Eating Disorder, Parasomnias, Somnambulism, Sleep-Related Smoking, Nocturnal Smoking, Topiramate

## Abstract

We describe a 45-year-old married woman with sleepwalking, sleep-related eating disorder, and sleep-related smoking behavior, but without restless legs syndrome. The patient had a history of mild obstructive sleep apnea with an AHI of 12.6/hour with an oxygen saturation nadir of 95%, which resolved after bariatric surgery. Treatment with topiramate 100mg at bedtime controlled all three parasomnias for ten months at the latest follow-up, with relapse occurring whenever topiramate was stopped. To our knowledge, this is the first reported successful treatment of sleep-related smoking. Clinicians are encouraged to inquire about other types of parasomnias, and other sleep disorders such as narcolepsy, in patients presenting with a complaint of one parasomnia.

## INTRODUCTION

Parasomnias are defined as undesirable physical events or experiences, and/or autonomic activity that occur during entry into sleep, during sleep, or during arousals from any stage of sleep. Parasomnias may occur during non-rapid eye movement (NREM) sleep, rapid eye movement (REM) sleep, or during sleep-wake transitions. Sleepwalking (SW) and sleep-related eating disorder (SRED) are NREM parasomnias that usually occur during N3 (slow-wave or deep sleep) with SW and during N2 sleep with SRED^1^. Sleep-related smoking (S-R smoking) was first reported in 2008 by Provini et al.^2^, who described six patients who complained of repeated awakenings with the desire to smoke and also eat due to “an inner drive”. Video-polysomnography (vPSG) in 3 of these cases documented an increased arousal index. A subsequent controlled study of 100 patients with restless legs syndrome (RLS) by Provini et al. (2010)^3^ found a significantly increased rate of S-R smoking in RLS patients compared to controls (12% vs. 2%). Furthermore, RLS patients with S-R smoking had significantly more frequent SRED than RLS patients without S-R smoking (83% vs. 26%). In addition, patients with narcolepsy type 1 were found to have increased rates of S-R smoking and SRED in a controlled study involving 65 patients and 65 controls, with increased rates of SRED (32% vs. 2%), S-R smoking (21% vs. 0%), and RLS (18% vs. 5%)^4^. To the best of our knowledge, treatment of S-R smoking has not been reported in the literature.

We now present a case of SW, SRED, and S-R smoking, but without apparent RLS, who responded to topiramate therapy.

## CASE REPORT

The patient is a 45-year-old married woman with a history of type 2 diabetes mellitus, hyperlipidemia, asthma, major depression, and chronic low back pain with a long-standing history of prescribed opiate use. The patient presented to the sleep clinic in November 2020 with the complaint of SW for two and a half years. These episodes initially occurred between 10 p.m. and 3 a.m. for one year, and subsequently, after 3 a.m. There was about 1 episode each night, which could last up to 1 hour. She reported waking up at places other than on her bed, including the floor, the edge of her bed, and sometimes in her bathroom or chair. If family members found her sleepwalking, they helped her return to her bed, and she would then resume sleep in her bed with no further SW episodes for that night. She would become very mean and rude, and disrespectful towards others during these episodes without subsequent recall. She reported never being awake during these episodes. There was no apparent precipitating factor for the onset of her symptoms.

The patient reported a history of chronic insomnia since 2000, with worsening insomnia since 2010, when she started working two jobs and reported sleeping for only 2-4 hours a night. She had previously tried trazodone and hydroxyzine with no benefit for her insomnia. There is no history of zolpidem use or other benzodiazepine receptor agonists. During her clinic visit in November 2020, the patient reported bedtime between 9:30 to 10:30 p.m. with a variable sleep latency of 15-120 minutes, with 0-3 nocturnal awakenings and a wake-up time of 4-4.30 a.m. The patient reported that she was sleeping 4-6 hours per night with one nap during the day for an hour, and the naps were restorative.

The patient also reported a history of sleep-related eating that started around the same time as her SW. She mentioned seeing empty food wrappers in her room but would not recall any of her eating behaviors the next day. She would eat any food available on her dresser, and if there was no food available in her room, there was no sleep-eating behavior. In addition, there were no episodes of hurting herself during the nocturnal eating episodes, and there was no episode of eating strange food as she mostly ate the snacks she had in her room. She also reported a history of “self-salvage,” depriving herself of food as soon as she would gain weight, but was never officially diagnosed with any daytime eating disorder.

The patient also reported a history of nocturnal smoking behavior. She noticed blisters on her feet due to stepping on lit cigarettes and spotted cigarette butts, carpet burns, and burnt bed sheets in her room, but could not recall any of the incidents the following day. Her husband would sometimes talk to her while she was smoking but would notice that she was “dazed” and only minimally awake and unaware of her surroundings. She denies any childhood history of parasomnias and no family history of parasomnias.

She had a history of mild obstructive sleep apnea documented by a sleep lab study in March 2012, with an AHI of 12.6/hour, and an oxygen saturation nadir of 95%. She then used CPAP for two years and stopped using it after undergoing gastric bypass surgery in May 2013 and then losing 124 pounds. The patient had two follow-up diagnostic sleep lab studies in July 2020 and in October 2020, which did not reveal any obstructive or central sleep apneas, periodic limb movements, hypoxemia, and no loss of REM atonia. In addition, the patient denied any history of RLS and had no family history of RLS.

The patient also has a history of chronic opiate use since 2002 for chronic low back pain and generalized body aches. From 2002 to 2016, the patient took hydrocodone 10mg 4 times a day and stopped taking it in 2016, and since then, she has been taking buprenorphine 8mg - 2-2.5 pills a day. She also has been taking gabapentin for her pain since 2010, 600mg in the morning and 1,200mg in the evening.

There was no history of sleep-driving. However, there was an episode where, after waking up at her usual early morning time and then driving to work, the patient fell asleep and hit a post shortly after starting to drive. There was no history of sleep-related sexual behavior and no violent behavior during her sleep, although once she fell and bruised her face while sleepwalking.

The patient has had a history of chronic depression since 1992. She took fluoxetine 30mg twice a day from 1992 to 2008 and then duloxetine 60mg twice a day and bupropion 100mg three times a day from 2008 to October 2020, followed by bupropion 450mg XL in the morning since October 2020. However, there was no improvement in nocturnal smoking with bupropion therapy. In addition, the patient reported smoking a pack of cigarettes a day since 2000.

Her medical history also included a history of migraine headaches. She also had febrile seizures when she was 1 or 2 years of age. There was no history of prior head injury or loss of consciousness. There is no history of meningitis, encephalitis, alcohol use, or illicit drug use. She has lived in the same house with her husband since 2011. The patient denies symptoms of restless leg syndrome, narcolepsy, nightmares, and dream enactment behavior. She worked as a medical coder since 2002 and stopped working in March 2020.

Based on clinical presentation, treatment was started with topiramate 25mg and slowly titrated up to 100mg at bedtime. The patient’s symptoms were not confirmed by a video polysomnography (vPSG). SW, SRED, and nocturnal smoking persisted at 25-75mg doses with complete resolution at 100mg. She reported missing one dose of topiramate on a single night and had an episode of SW and talked over the phone that night, of which she was unaware. There were no reported side effects with topiramate, and the patient reported sleeping for 8 hours a night for the initial three weeks since starting topiramate therapy. The patient subsequently followed up in the sleep clinic for ten months and reported no further SW, SRED, and S-R smoking episodes.

## DISCUSSION

NREM parasomnias, including disorders of arousal and SRED, are often provoked or exacerbated by various factors. The common exacerbating factors of SW include sleep deprivation and physical and emotional stress^5^. This patient has a history of chronic insomnia, and the total nocturnal sleep time reported by the patient was variable between two and six hours, which indicates chronic partial sleep deprivation. In addition, stress and a history of migraines in this patient may have contributed to SW as well.

The usual age of onset of NREM parasomnias is in childhood. However, an interesting fact was that this patient developed SW, SRED, and S-R smoking after age 40. Recurrent sleepwalking episodes and prolonged incomplete sleep paralysis have been reported in a man with onset after 40 years of age^6^. In addition, based on a study of 100 patients with repeated nocturnal injury, late-onset night terrors and SW were reported to occur as late as age 58 years among 54 patients; 33% of these patients had SW and night terrors emerge after age 16^7^.

Treatment with topiramate was initiated in this case, given its documented benefit for controlling SRED in several reports^8^. Most notably, in a randomized control trial, SRED symptoms were significantly reduced with topiramate (74.7% to 33.2% nights/week; n=15) compared to placebo (77.0% to 57.4%; n=17)^9^.

The literature offers some evidence for how topiramate can control sleep-related smoking as an addictive disorder and sleep-related eating disorder as a compulsive behavior disorder within the spectrum of other addictive and compulsive disorders. In a randomized, double-blind, placebo-controlled study, topiramate was reported to reduce cocaine’s abuse liability, presumably through antagonism of excitatory glutaminergic pathways and facilitation of inhibitory gamma-aminobutyric acid neurons in the cortico-mesolimbic system, as discussed by the authors^10^. In a systematic review of topiramate therapy for a spectrum of addictive and eating disorders, efficacy was greatest for alcohol abuse, followed by binge eating disorder and cocaine use disorder^11^. Topiramate has also been reported as successful therapy in reported cases of compulsive buying disorder^12^, compulsive gambling^13^, and also as successful therapy of compulsive sexual behavior in a case report^14^.

After ten months of follow-up, the patient did not report further SW or SRED episodes while on topiramate. In addition, there were no further S-R smoking episodes.

Sleep-disordered breathing is another known exacerbator of SW, but this patient’s OSA had resolved with bariatric surgery. Hence, sleep-disordered breathing was not an issue in this patient.

Our patient also has had a history of major depression since age 17, with prior fluoxetine, duloxetine, and bupropion therapy. Bupropion has been associated with triggering de novo SW^15^. However, the patient had sleepwalked before using this medication, and the initiation of bupropion therapy did not exacerbate the SW.

Bupropion has proven efficacy for smoking cessation in several clinical trials. For example, an open-label randomized control trial of 1,071 patients found significantly improved abstinence rates for smoking with bupropion (27.9%) when compared to nicotine replacement therapy (NRT) (24.2%) or a combination of NRT and bupropion (24.2%). Bupropion was also more beneficial than NRT in those with a history of depression (29.8 versus 18.5%)^16^. However, our patient showed no improvement in daytime smoking or S-R smoking with bupropion therapy despite high-dose (450mg) therapy.

Also, prior therapy with hydrocodone and then current therapy with buprenorphine and gabapentin may have been masking any underlying RLS symptoms, and so it is possible that this patient may have also had at least mild RLS. However, the lack of PLMs during her vPSGs, the lack of prior RLS symptoms before starting opiate and gabapentin therapy, and the lack of a family history of RLS make the diagnosis of RLS relatively unlikely. So, from this perspective, this case of combined SRED and S-R smoking, without associated RLS, is atypical.

This appears to be the first reported case of successful therapy of S-R smoking, consisting of bedtime topiramate that also controlled SRED and SW.

In conclusion, S-R smoking can be associated with SRED and SW, as illustrated in this case. Therefore, clinicians who evaluate patients with SW and other parasomnias should inquire about other forms of NREM parasomnias, including confusional arousals, sleepwalking, sleep terrors, sleep-related eating, sleep-related sexual behaviors, sleep-related smoking, and also any relevant psychiatric history. Topiramate was shown to be beneficial in the management of SW and S-R smoking, besides SRED. However, further research is needed to identify the etiology, predisposing and precipitating factors, and pharmacological interventions to manage S-R smoking.
